# RETRACTION: Rapid Rehabilitation Effect on Complications, Wound Infection, Anastomotic Leak, Obstruction, and Hospital Re‐Admission for Gastrointestinal Surgery Subjects: A Meta‐Analysis

**DOI:** 10.1111/iwj.70340

**Published:** 2025-03-17

## Abstract

**RETRACTION:** LiuL. , HeL. , QiuA. , and ZhangM. , “Rapid Rehabilitation Effect on Complications, Wound Infection, Anastomotic Leak, Obstruction, and Hospital Re‐Admission for Gastrointestinal Surgery Subjects: A Meta‐Analysis,” International Wound Journal 19, no. 6 (2022): 1539–1550, 10.1111/iwj.13753.35191597 PMC9493214

The above article, published online on 22 February 2022, in Wiley Online Library (http://onlinelibrary.wiley.com/), has been retracted by agreement between the journal Editor in Chief, Professor Keith Harding; and John Wiley & Sons Ltd. Following an investigation by the publisher, all parties have concluded that this article was accepted solely on the basis of a compromised peer review process. The editors have therefore decided to retract the article. The authors did not respond to our notice regarding the retraction.

**RETRACTION:** L. Liu , L. He , A. Qiu , and M. Zhang , “Rapid Rehabilitation Effect on Complications, Wound Infection, Anastomotic Leak, Obstruction, and Hospital Re‐Admission for Gastrointestinal Surgery Subjects: A Meta‐Analysis,” International Wound Journal 19, no. 6 (2022): 1539–1550, 10.1111/iwj.13753.35191597 PMC9493214

The above article, published online on 22 February 2022, in Wiley Online Library (http://onlinelibrary.wiley.com/), has been retracted by agreement between the journal Editor in Chief, Professor Keith Harding; and John Wiley & Sons Ltd. Following an investigation by the publisher, all parties have concluded that this article was accepted solely on the basis of a compromised peer review process. The editors have therefore decided to retract the article. The authors did not respond to our notice regarding the retraction.

